# Plasticity of collective behavior in a nomadic early spring folivore

**DOI:** 10.3389/fphys.2013.00054

**Published:** 2013-03-22

**Authors:** Emma Despland

**Affiliations:** Department of Biology, Concordia UniversityMontréal, QC, Canada

**Keywords:** *Malacosoma disstria*, foraging, thermoregulation, Lepidoptera, decision-making, group-living, social behavior

## Abstract

Collective behavior in the forest tent caterpillar (*Malacosoma disstria*) meets the thermal constraints of being an early spring folivore, but introduces other constraints in food choice. These are minimized by state-dependent, inter-individual, and ontogenetic variations in responses to social cues. Forest tent caterpillars use pheromone trails and tactile communication among colony members to stay together during foraging. At the group level, these rules lead to cohesive synchronized collective nomadic foraging, in which the colony travels en masse between feeding and resting sites. This paper proposes that synchronized collective locomotion prevents individuals from becoming separated from the colony and hence permits them to reap the advantages of group-living, notably collective basking to increase their body temperature above ambient and collective defense against natural enemies. However, this cohesive behavior also implies conservative foraging, and colonies can become trapped on poor food sources. High fidelity to pheromone trails leads to strong amplification of an initial choice, such that colonies seldom abandon the first food source contacted, even if a better one is nearby. The risk of this trapping is modulated both by consistent inter-individual variations in exploratory behavior and by inner state. Colonies consisting of active-phenotype or protein-deprived individuals that explore more-off trails exhibit greater collective flexibility in foraging. An ontogenetic shift toward more independent movement occurs as caterpillars grow. This leads to colony break-up as the season advances. Selection pressures facing older caterpillars favor solitary living more than in the earlier instars. Caterpillars respond to this predictably changing environment by altering their behavioral rules as they grow.

## Early spring caterpillars as social organisms

### Physiological requirements of caterpillars

Caterpillars, like other larvae, are faced with the challenge to grow and develop as fast as possible, while avoiding other hazards such as predation and disease. Indeed, adult size is strongly correlated with fecundity, and rapid development implies spending less time in the vulnerable larval stage (Parry et al., 1998). For instance, a typical forest tent caterpillar [*Malacosoma disstria* (Lasiocampidae)] grows from <1 to 300–600 mg within ~6 weeks (Fitzgerald, 1995). As herbivores, caterpillars face the additional challenge that this high growth rate must be achieved on a poor, and in particular a protein-limited, food source, namely leaves. This is achieved by consuming huge quantities of food. Indeed, caterpillars generally eat about 10 times their body weight daily. Their daily time budget is therefore almost entirely taken-up by consuming, then digesting, this food.

This paper examines early spring feeding and nomadic group-living as strategies used by caterpillars to maximize growth and minimize risk. In particular I use the forest tent caterpillar as a model system to study social communication in the context of nomadic foraging as a behavioral adaptation to the environmental constraints of being an early spring feeding caterpillar.

### Early spring feeding caterpillars

Many Lepidopterans maximize their larval growth rate by timing their life cycle to feed on the higher quality foliage available in the spring. Indeed, in the weeks following budbreak, the foliage of most temperate deciduous trees contains more nitrogen and fewer defensive compounds than it does later in the summer (Hunter and Lechowicz, 1992). Early spring feeders synchronize hatching with leaf flush to focus on this narrow window of opportunity. A delay of even a week can significantly reduce caterpillar growth rates and retard development (Jones and Despland, 2006).

Hatching early in spring allows caterpillars to feed on higher quality foliage, in particular to accumulate more growth-limiting protein. It also permits caterpillars to develop in an enemy-free space before predators and parasitoids become active (Parry et al., 1998). However, it implies the drawback that caterpillars must hatch and begin to feed long before temperatures become favorable for physiological processes.

Indeed, as ectotherms, caterpillars perform best at warmer temperatures and show limited ability to regulate their body temperature distinctly from ambient. For instance, for the forest tent caterpillar optimal growth occurs at 25°C (Lévesque et al., 2002) but spring temperatures experienced by this species are often much lower: in one study, daytime temperatures experienced by second instar caterpillars varied between 12 and 26°C and nights were too cold to permit foraging (McClure and Despland, 2010). Early spring feeders therefore often experience growth limitation by temperature.

These caterpillars often show an adaptive suite of behavioral and anatomical traits to increase their body temperature (Casey, 1993): many early season caterpillars seek out warmer microhabitats and orient their bodies toward the sun for basking, and they are dark-colored and thickly covered with setae to reduce convective heat loss (Bryant et al., 2000).

However, their small body size limits the thermal gains attained by solitary caterpillars (Joos et al., 1988; Bryant et al., 2000; Ruf and Fiedler, 2000), and many early spring species increase body temperature by living communally (Stamp and Bowers, 1990b; Klok and Chown, 1999; Bryant et al., 2000), in some cases even building silk tents that trap solar heat (Casey et al., 1988; Joos et al., 1988; Ruf and Fiedler, 2000, 2002b). Indeed, thermal constraints have been postulated as a major driver of group-living in caterpillars.

### Group-living caterpillars

Larvae that stay aggregated during at least part of their development and exhibit collective behavior are observed in over 300 Lepidopteran species spread across 20 families, and group-living is thought to have evolved several times within the order (Costa, 2006). Many female moths lay their eggs in batches, and upon hatching, the larvae disperse to forage independently in some species, but in others, they aggregate and remain together for at least part of their development. So caterpillar colonies generally consist of an aggregation of siblings that use various signals to communicate and perform social behaviors but eventually disperse at some point in their development before reaching the adult stage.

Caterpillar colonies are often observed feeding and resting together. Their collective behavior is characterized by synchronized group movement. Social caterpillars often march in long processions using a combination of tactile cues from neighbors and trail-based cues, and even in some cases acoustic cues (Fletcher et al., 2006). Trails are made of a fine thread of silk extruded from the spinnerets of each passing caterpillar; in many species, this trail is overlaid by a pheromone, and it is the latter that elicits the following behavior, the silk serving mainly for the caterpillars to grip the surface (Fitzgerald and Edgerly, 1979; Fitzgerald and Underwood, 1998b; Fitzgerald and Pescador-Rubio, 2002; Fitzgerald, 2003). Other collective behaviors often observed in caterpillar colonies include group basking, collective defense against predators or parasitoids, shelter building, and collective feeding to overwhelm plant defenses (Fitzgerald, 1993; Costa and Pierce, 1997; Fitzgerald and Costa, 1999; Costa, 2006).

Caterpillar societies are traditionally classified by foraging type (Costa, 1997), as this generally determines the forms of social communication exhibited [see Costa (2006) for a detailed summary of social behavior in all species investigated so far]. Some are patch-restricted, which build a shelter on the food plant and feed inside it, gradually increasing its size. This is the simplest form of social organization seen in caterpillar colonies, and communication between colony-mates occurs primarily via construction of the shelter. Many other species are nomadic, and move en masse between food sources and resting sites. Organization of collective locomotion in these species resembles that seen in migrating ungulate herds or fish schools (Couzin and Krause, 2003), where individuals use communication to remain together during travel. In this situation, synchronization of behavior is essential to maintain group cohesion, and therefore a collective decision must arise about the timing and direction of travel (Conradt and Roper, 2005; Jeanson et al., 2012). I will examine both the constraints that this imposes and the mechanisms providing flexibility in collective decisions, based largely on recent research on a nomadic foraging caterpillar, the forest tent caterpillar.

Finally, some species are central-place foragers, building a communal shelter from which individuals, either alone or in groups venture forth to feed on food patches, returning to the shelter afterwards. It is among the central-place foragers that the most sophisticated social communication is observed, including collaborative foraging by which individuals use silk-and-pheromone trails to recruit colony-mates to food finds, in some cases using differential signaling according to patch quality. This altruistic signaling is thought to be favored by kin selection since colony-mates are usually siblings. The nest thus acts not only as a shelter but also as a communication center, which, with the network of trails, channels the information that directs colony behavior (Fitzgerald and Costa, 1999), via elaborate social signaling similar to that observed in eusocial insects [see for example (Fitzgerald and Peterson, 1983; Fitzgerald and Underwood, 1998a; Ruf et al., 2003)].

We focus on nomadic foragers, which, although very numerous, have received considerably less attention to date than the central-place foragers (Costa, 2006), and propose that, in these species, trail-use serves mainly to keep the group together during locomotion rather than to improve individual foraging success by recruitment to high-quality food patches as seen in central-place foragers. Therefore, in order to understand the selection pressures shaping collective nomadic foraging, one must first ask why grouping is adaptive. Several different advantages have been suggested for aggregation in caterpillars, including anti-predator defense, feeding facilitation and thermoregulation (Fitzgerald, 1993; Costa and Pierce, 1997). The relative importance of these benefits naturally depends on the species' ecology and life history. For instance, grouping does not confer thermal benefits in tropical *Doratifera casta* (Limacodidae) (Reader and Hochuli, 2003), but does in the temperate-zone early spring feeding New England buckmoth *Hemileuca lucina* (Saturniidae) (Stamp and Bowers, 1990b).

We propose that thermoregulation is a key selection pressure that has shaped the collective behavior of an early spring feeding nomadic forager, the forest tent caterpillar, and that this leads to conservative foraging [i.e., low collective flexibility sensu Fitzgerald and Costa (1999)]. In this context, responses to social cues mediate the trade-off between staying with the group to reap thermal benefits and exploring new territory to discover new food sources, and these responses are modulated by individual physiological state, behavioral phenotype and age. First, I examine the mechanisms of social communication that keep colonies together, then discuss the role of collective anti-predator defense and thermoregulation as possible selection pressures favoring grouping. I next discuss consequences of synchronized collective locomotion for food choices and examine mechanisms of flexibility that modulate behavior in the face of varying resource quality and changing selection pressures as caterpillars grow.

## The forest tent caterpillar: a group-living nomadic early spring feeder

### Ecology and life history

The forest tent caterpillar is an outbreaking pest of deciduous trees across much of North America. Populations undergo irregular cycles, growing to extremely high densities and defoliating entire stands, then crashing abruptly as a result of poor weather, parasitoids, and epizootic viral disease (Fitzgerald, 1995).

The forest tent caterpillar uses different host trees in different parts of its distribution range (Parry and Goyer, 2004). It is an early spring feeder. In eastern Canada, hatching date is generally synchronized with budbreak of the primary host, trembling aspen (*Populus tremuloides*), one of the earliest trees to leaf-out in spring. For example, one study in southern Québec records that caterpillars hatched on May 21st when daytime temperatures were between 2 and 13°C (McClure and Despland, 2010).

Caterpillars moult through five instars in approximately 6 weeks, gradually becoming more independent, then pupate singly. The adults that emerge in mid-summer do not feed and live only a few days. The females mate once and lay their full complement of eggs in a band around a host tree twig. The eggs overwinter and larvae emerge the following spring (Fitzgerald, 1995).

The forest tent caterpillar exhibits nomadic foraging. Upon emergence from the egg-band, siblings aggregate in a colony and build a silk mat as a temporary bivouac; they then alternate between feeding and returning to the bivouac to digest between meals. Colonies are highly cohesive in early instars and rarely fragment; their activity is highly synchronous, with most of the colony generally moving together (Fitzgerald and Costa, 1986; McClure and Despland, 2010). The rate of alternation between feeding and digesting bouts depends on temperature, presumably based on thermal effects on the physiological processes of walking, feeding and digestion (Peters and Despland, 2006). Foraging slows but continues down to 10°C and can occur at any time of day (or night) if temperature permits (McClure and Despland, 2010). On high-quality food, the colony generally returns to exploit the same food source at each foraging bout until it is depleted, but on lower quality hosts they switch more often between food sources. Similarly, they tend to return to the same bivouac after feeding and only abandon a bivouac after a molt, when the bivouac becomes too soiled with exuviae (Fitzgerald and Costa, 1986; McClure and Despland, 2010). As the caterpillars grow, they gradually forage independently more often, but still return to the bivouac to bask together.

Remaining with a group is vital to young forest tent caterpillars. Indeed, isolated early instar larvae die, even under ideal laboratory conditions (Robison, 1993). Young isolated caterpillars feed less than those in groups, even in lab experiments when easily accessible artificial diet is provided, but spend more time searching for a pheromone trail and wandering (Despland and Hamzeh, 2004), presumably looking for a group to rejoin (Klok and Chown, 1999). The next section describes the underlying responses to the abiotic environment and to social cues from colony-mates.

### Social communication

Like many social caterpillars, moving forest tent caterpillars spin a fine thread of silk and mark it with a trail pheromone. The pheromone is not very volatile (i.e., it is long-lasting), and neither quantity nor quality of trail marking depends on whether the individual has fed: all individuals in a procession lay down the silk and mark it, both on the way to and back from feeding (Fitzgerald and Costa, 1986). Caterpillars of all developmental stadia preferentially follow this pheromone rather than move over unmarked territory, prefer fresh over older trails and travel faster when trails are present. Younger caterpillars also exhibit leader following and, when trails are absent, generally follow a departing colony-mate (Colasurdo and Despland, 2005). Caterpillars exhibit a stereotyped behavior called searching, which involves swaying the head from side-to-side and brushing the labial palps on the substrate to detect trail pheromone. In the absence of trails, caterpillars are less likely to initiate locomotion and tend to engage in searching rather than directed walking. When a colony-mate is present, active caterpillars are more likely to become quiescent, settling down to rest beside it (Despland and Hamzeh, 2004). Compared to older caterpillars, younger caterpillars are less active and less likely to either leave a colony-mate or move onto unmarked ground (Despland and Hamzeh, 2004; Colasurdo and Despland, 2005).

These observations imply that trail use is a key component of forest tent caterpillar locomotion. When trails are present, caterpillar colonies find food sources much faster: one study showed that, in laboratory arenas, second-instar colonies discovered a nearby food source within 2 h when trails were provided, but in the absence of trails most groups had not found the source after 4 h (Despland and Le Huu, 2007). Trails both decrease the latency to initiate locomotion and increase the speed of travel. In the absence of trails, the latency to initiate locomotion decreases with increasing group size (McClure et al., 2013).

Young forest tent caterpillars thus rely strongly on social cues, both pheromone trails and contact with colony-mates, to initiate and direct locomotion, to an extent that can seem surprising, for instance, when an isolated young caterpillar is unable to find and exploit a nearby food source and dies as a result. I posit that the evolutionary function of this form of communication is to keep the group together in order to reap the advantages of group-living, including anti-predator protection and thermoregulation.

## Advantages of collective behavior in the forest tent caterpillar

### Collective anti-predator defense

A general review shows lower predation mortality rates in gregarious than in solitary caterpillars, and a variety of mechanisms have been proposed (Hunter, 2000). The simplest possible explanation for increased anti-predator protection in groups is simple dilution (fewer individuals are taken from a group due to predator saturation) and the selfish herd effect (individuals in the center of a group are less likely to be captured than those on the edges). Forest tent caterpillar colonies have been shown to benefit from both these effects (McClure and Despland, 2011) when attacked by invertebrate predators (spiders, stinkbugs, parasitic wasps). Spiders and stinkbugs can only take one prey item at a time, and so in a large group, individual rate of capture is lower. All three natural enemies examined were more likely to attack an individual on the edge of a group than one in the center. However, dilution is ineffective against collective foraging predators that can rapidly recruit colony-mates to decimate a caterpillar colony [e.g., vespid wasps (Stamp and Bowers, 1991; McClure and Despland, 2010)].

For brightly colored insects that are toxic to predators, there is considerable evidence showing that the warning colors are more effective at deterring predators when the insects are aggregated (Tullberg et al., 2000; Hatle and Salazar, 2001). Indeed, warning coloration and repellent defences are particularly common in gregarious insects. The forest tent caterpillar is typical in that it is brightly colored and groups certainly are conspicuous, and it is distasteful to birds (Heinrich, 1983, 1993), but the relationship between grouping, coloration, chemical defense, and predation is hard to prove. Other group-living caterpillars that might benefit from collective enhancement of the aposematic signal include *Uresiphita reversalis* (Pyralidae) (Bernays and Montllor, 1989), *D. casta* (Limocodidae) (Reader and Hochuli, 2003), *Pryeria sinica* (Zygaenidae) (Costa, 2006), *Saucrobotys futilalis* (Crambidae) (Grant, 2007).

Grouped caterpillars can also use collective behavior to decrease both attack rate and individual investment in anti-predator behavior, using shared vigilance and/or collaborative defense. Many gregarious caterpillars exhibit synchronized head-flicking in response to an approaching flying predator or parasitoid (Myers and Smith, 1978; Reader and Hochuli, 2003), and some, such as the Eastern tent caterpillar (*Malacosoma americanum*, Lasiocampidae), combine these displays with defensive regurgitation (Peterson et al., 1987). Others [including *Phalera bucephala* (Notodontidae), several *Datana* (Notodontidae) and *Neodiprion* (Symphyta) species] display a defensive U-shape, raising the tip of the abdomen and arching the head and thorax back, and regurgitate if the threat amplifies (Costa, 2006). These defensive behaviors radiate rapidly through a group, suggesting some form of shared vigilance, whereby individuals become aware of the presence of a predator via the behavior of their neighbors. Tactile and visual cues, as well as vibrations transmitted through the silk net to which the caterpillars cling, have been suggested as possible modes of transmission (Fitzgerald, 1993). Head-flicking has been shown to be effective as a collaborative defense against parasitic wasps in the forest tent caterpillar (McClure and Despland, 2011).

Collective defense has often been suggested as a benefit to group-living in caterpillars, but seldom tested [but see McClure and Despland (2011)]. Moreover, the predation pressure exerted on these caterpillars is not clear, particularly for early spring feeders that are active earlier in the season than most predators (Parry et al., 1998). It is therefore difficult to evaluate the strength of collective defense as a selection pressure favoring group-living in caterpillars.

### Collective thermoregulation

Collective thermoregulation is another often cited advantage to group-living in caterpillars. Indeed caterpillars in groups can attain higher temperatures by basking than can isolated individuals, as observed in several temperate-zone early spring feeders (Porter, 1982; Stamp and Bowers, 1990a; Bryant et al., 2000), and a South African bivoltine species (Klok and Chown, 1999). Caterpillars huddled in tight aggregations decrease their exposed surface area and thus reduce convective and evaporative heat loss and conserve metabolic heat (Seymour, 1974). By the same mechanism, they can also lower water loss (Klok and Chown, 1999).

In the forest tent caterpillar, group-living and synchronized behavior is essential to reap the thermal gains of basking: under field conditions, tightly aggregated groups can increase their temperature significantly above ambient by basking, but isolated individuals cannot (McClure et al., 2011a). Collective basking permits caterpillars to maintain their body temperature close to optimal (25°C) and hence to improve their physiological performance. Indeed, both food consumption and the proportion of food converted to biomass increase up to 25°C, leading to increase in growth and acceleration of development (Lévesque et al., 2002).

Collective thermoregulation has clearly been shown to be a major advantage of shelter use in central-place foraging caterpillars. Colonies return to their shelter between foraging bouts and use it to maintain optimal temperatures and maximize food processing during quiescent bouts to ensure gut-emptying before the next foraging bout. Indeed, several temperate-zone early spring species have been shown to be able to closely regulate body temperature and improve growth through use of different microhabitats in the shelter [e.g., the Eastern tent caterpillar (Joos et al., 1988), and the small Eggar moth (*Eriogaster lanestris*, Lasiocampidae) (Ruf and Fiedler, 2002b)]. Both the pine processionary caterpillar [*Thaumatopoiea pityocampa* (Thaumetopoeidae)] in the Mediterranean and the madrone caterpillar [*Eucheira socialis* (Pieridae)] in the Sierra Madre Occidentalis of Mexico use a shelter to continue foraging during winter when temperatures drop below freezing (Fabre, 1899; Fitzgerald and Underwood, 2000). Shelter-based thermoregulation is thought to be one of the key drivers shaping collective behavior in central-place foragers, including not only shelter construction but also synchronization and timing of foraging forays outside the shelter (Fitzgerald, 1993).

Even in the absence of a shelter, improved temperature and water regulation through collective basking appears to be a significant advantage to group-living for nomadic foragers as well, including the forest tent caterpillar (McClure and Despland, 2010), the anomalous Emperor moth *Imbrasia belina* (Saturniidae) (Klok and Chown, 1999) and the Australian sawfly *P. affinis* (Symphyta) (Fletcher, 2009). For instance, in the New England buckmoth, larvae constrained by predation to forage alone in the shade grow poorly, whereas in the absence of predation, larvae remain aggregated, bask collectively in the sun, select high-quality young leaves and grow twice as fast (Stamp and Bowers, 1990b). The next section develops the hypothesis that collective thermoregulation is an important driver of collective behavior in nomadic species as well as in tent-users, and examines the extent to which thermal constraints shape spatial and temporal patterns of collective nomadic foraging.

## Abiotic environment modulates collective behavior

### Thermal environment

The forest tent caterpillar not only benefits from collective thermoregulation, it also exhibits patterns of collective behavior that emerge from individual responses to temperature. Indeed, the rhythm of alternation of collective feeding and digesting bouts accelerates at higher temperature (Peters and Despland, 2006), as interactions within a colony synchronize individual foraging schedules (Despland and Simpson, 2006). The speed at which individual caterpillars walk, eat, and digest their food depends on ambient temperature (Fitzgerald, 1995), which leads to shorter foraging and digesting bouts as temperature increases (Peters and Despland, 2006).

Temperature effects on foraging schedules have been studied in three central-place foragers: the small Eggar moth shows a pattern similar to that described here for the forest tent caterpillar, with foraging accelerating at higher temperature (Ruf and Fiedler, 2002a), whereas the Eastern tent caterpillar (Casey et al., 1988; Fitzgerald et al., 1988) and the madrone caterpillar (Fitzgerald and Underwood, 2000) show fixed circadian foraging schedules. In both cases, synchronized collective foraging is a self-organized collective phenomenon (Fitzgerald and Visscher, 1996) that arises from individuals' responses to the abiotic environment, to their own internal state and to cues from colony-mates; however, in the first case (thermally flexible schedule), responses to temperature effects on internal state play an important role in determining when to forage, whereas in the second (fixed schedule) they do not, possibly because they are mitigated by use of the tent. Reliance on temperature-based internal state cues to direct foraging, as shown by the forest tent caterpillar and the small Eggar moth leads to the emergence of a flexible schedule that varies according to thermal environments and matches individual physiological requirements while keeping the colony together (Peters and Despland, 2006).

Not only the temporal, but also the spatial pattern of forest tent caterpillar collective foraging appears to depend on thermal considerations. Individual forest tent caterpillars move-up temperature gradients up to 30°C, where they switch to thermo-negative behavior and hang from their abdominal prolegs in the shade (Fitzgerald, 1995). When a radiant heat source is provided at a distance from the food, caterpillars behave to maximize thermal gains: colonies move away from the food to bivouac under the heat source, spend more time on the bivouac and cluster in a more cohesive group (McClure et al., 2011a). Thus, microenvironment gradients of temperature within a host tree can determine the location of bivouacs: forest tent caterpillar colonies in the field are observed in the early morning marching out of the tree canopy to bask on the south-east side of the trunk.

Thermal needs thus influence activity schedule, colony aggregation, and habitat selection in the forest tent caterpillar. These emergent group-level properties result from individual-level responses to internal, social and thermal cues. Indeed, individual locomotion is modulated by temperature, leader following and trail fidelity ensure that groups move together, and responses to thermal gradients lead groups to move to thermally appropriate microenvironments. These mechanisms ensure that the direction and schedule of movement matches the thermal conditions, and that the colony stays together in order to continue to reap the thermal gains of collective basking. The next section examines the consequences of this cohesive collective foraging behavior for food choice and the role played by the quality of food sources in directing collective foraging.

### Nutritional environment

Fabre (1899) was the first to record the potential nutritional pitfalls associated with strong leader following and fidelity to pheromone trails: he observed a colony of pine processionary caterpillars that had formed a circular trail around the edge of a flower pot. They became trapped on that trail, following each other around in circles and failing to discover a nearby food source. Activity ceased overnight when temperatures dropped, but circling resumed each morning. On the third morning, the lead caterpillar found itself slightly off the trail and moved-off onto the unexplored territory inside the flower pot. It was followed by 6 colony-mates, but a break in the chain implied that the seventh follower, not in physical contact with the preceding individual, remained on the stronger trail around the edge rather than the weaker one down the inside. This small group, finding nothing inside the flower pot, returned to the edge and resumed circular marching. Finally, on the 8th day, a caterpillar stuck behind an unmoving colony-mate moved-off the trail, down the outer edge of the pot. The individuals behind it followed and the group soon relocated to a pine branch at the base of the pot (Fabre, 1899). Fabre concluded that “disorganization” of the procession caused by cold, weakness and starvation was required to break the rigid pattern of trail-following.

More recently, forest tent caterpillar colonies in choice tests have been shown to become trapped on the first food source contacted, even if it is of low quality, unable to relocate to a higher quality source nearby (Dussutour et al., 2007). As the caterpillars leave the bivouac, they choose a direction at random (in the homogeneous environment of the laboratory) and a trail is formed to the first food source contacted. As more caterpillars use the trail, it becomes reinforced and the probability of leaving decreases. Even if a few individuals leave the well-marked trail and discover a better food source, the rest of the group stays on the older better-marked trail (Dussutour et al., 2007).

These two studies show how too strong amplification, in the form of high-fidelity trail-following, can lead groups to become trapped on suboptimal choices. In many social organisms (including eusocial hymenopterans), this is avoided by quality-dependent amplification via differential signaling, for example when the strength of the pheromone trail deposited depends on the quality of the resource (Jeanson et al., 2012). This type of sophisticated differential recruitment is shown by some central-place foraging caterpillars such as the Eastern tent caterpillar (Fitzgerald and Peterson, 1983) and the small Eggar moth (Ruf and Fiedler, 2002a), but not by others [madrone caterpillar (Fitzgerald and Underwood, 1998b), *Gloveria* spp. (Fitzgerald and Underwood, 1998a)]. This leads to differences in flexibility of collective foraging: the Eastern tent caterpillar forages selectively on high-quality food sources whereas the madrone caterpillar can be trapped on poor sources (Costa, 2006). Like the madrone caterpillar and *Gloveria* spp., forest tent caterpillars overmark a trail each time they pass on it and trail strength thus increases with traffic; however, they do not adjust their mark according to their satiety state and hence do not transmit information about food quality when marking a trail (Fitzgerald and Costa, 1986). Trail-followers thus prefer trails laid by larger than by smaller groups, but cannot distinguish between the trails of fed and satiated individuals (Fitzgerald and Underwood, 1998a; McClure et al., 2013) and do not easily relocate to new and better food sources (Fitzgerald and Underwood, 1998a; Dussutour et al., 2007).

The nutritional environment thus does not seem to influence production of social signals in the forest tent caterpillar, as it does in some central-place collective foragers; however, it does appear to influence individuals' responses to those cues. Hungry, and specifically protein-deprived, caterpillars are more likely to leave resting colony-mates, to initiate locomotion and to move-off of a trail onto unexplored territory (Colasurdo et al., 2007). Therefore, within colonies, hungry caterpillars are more likely to initiate movement and lead moving groups than sated individuals, and colonies with a high proportion of unfed caterpillars initiate foraging sooner than those comprising mainly sated individuals (McClure et al., 2011b). Although colonies can become trapped on low-quality protein-containing food sources (Dussutour et al., 2007), they more rapidly abandon low-quality protein-poor food sources and initiate exploration (McClure et al., 2013).

These experiments show that differential recruitment is not necessary for a group to collectively choose the better of two alternatives. Indeed, in many of these collective choice trials, the entire colony relocated en-masse to the new source and no individuals returned to recruit colony-mates after having sampled the new source (McClure et al., 2013). Instead, the choice can emerge from differences in retention at patches of different qualities, based on individual nutritional state. This decrease in responsiveness to cues associated with protein deprivation implies an increase in noise in social communication, and hence these results support the idea that a certain level of noise can be adaptive as it increases flexibility in collective decisions (Jeanson et al., 2012). Indeed, it was the “disorganization” resulting from starvation, fatigue and cold that Fabre (1899) cited as the mechanism that allowed the pine processionary caterpillars to break free of the constant circling around the flower pot rim.

These findings underscore the multivariate nature of food and the complex ways in which food quality affects behavior, since it is protein deprivation specifically, that promotes exploration. Indeed, protein is usually the main deficient nutrient for herbivores. Caterpillars, and other larval folivorous insects, are exceptional in achieving very high growth rates on the very protein-poor food constituted by leaves (White, 2005). They therefore generally exhibit strong preferences for foods containing high protein, are efficient in post-ingestive use of dietary protein and perform better on more protein-rich foliage [see for example Lindroth and Bloomer (1991) and Lévesque et al. (2002)].

Few studies have examined the effects of internal state on movement (Holyoak et al., 2008). Nonetheless it appears that animals can alter foraging behavior in response to nutrient deficiency in order to increase the probability of encountering new, and possibly more nutritious, food sources. Notably, insects including caterpillars increase locomotion on poor diets (Barton-Browne, 1993; Nagata and Nagasawa, 2006). Protein seems to be a key nutrient triggering this increased locomotion, rather than simply poor food: in locusts, increased locomotion is observed in response to falling levels of haemolymph amino acids (Abisgold and Simpson, 1987). The forest tent caterpillar shows increased locomotion and switching between food sources on poor quality host plants (Etilé, 2008; McClure and Despland, 2010), and lab experiments show that this is triggered specifically by protein deprivation (Colasurdo et al., 2007).

Recent studies have also suggested that protein limitation affects collective locomotion of insect groups more than the movement of individuals (Bazazi et al., 2011). Indeed, protein deprivation has been shown to drive the formation and mass migration of large bands of three different locust or cricket species (Simpson et al., 2006; Bazazi et al., 2008; Hansen et al., 2011). This occurs because, in locusts as in forest tent caterpillars, protein limitation affects not only individual locomotion but also responses to social cues. However, the nature of the effect is very different: in locusts, protein deprivation leads to higher interaction strength (as individuals seek both to cannibalize their neighbors and to avoid being eaten themselves) and hence to the emergence of more coherent group movement (Bazazi et al., 2011). By contrast, in the forest tent caterpillar, protein deprivation decreases interaction strength and hence group cohesion (Colasurdo et al., 2007; McClure et al., 2013).

Thus, strong colony cohesion, likely driven by thermoregulatory needs, leads to very conservative foraging in the forest tent caterpillar, also known as low collective flexibility (Fitzgerald and Costa, 1999). Differences in collective flexibility have been documented between species in two taxa of central-place foraging eusocial insects, namely ants (Beckers et al., 1990) and stingless bees (Schmidt et al., 2006), and, as discussed above for central-place foraging caterpillars, they are tied to forms of recruitment. In general, it appears that species that use differential recruitment based on source quality exhibit high collective flexibility and switch rapidly to newly discovered richer sources, whereas low collective flexibility is observed when signals do not vary with patch quality (Beckers et al., 1990; Fitzgerald and Underwood, 1998a; Schmidt et al., 2006). I show that, in the forest tent caterpillar, collective flexibility is low but not nil, and that it arises from differences in retention at a source due to modulation of individual responses to social signals based on protein satiation.

Nomadic foragers in general use various social signals to maintain cohesion during collective choices, including not only pheromone trails, but also physical contact between individuals (e.g., pine processionary caterpillars), vision (e.g., schooling fish) and acoustic calls (e.g., sawfly larvae, birds and primates)—(Fletcher, 2008). Responding to these cues is a form of allomimesis and amplifies initial choices; lowered responsiveness can lead to a decrease in retention at a source. Such differences in retention have been shown to lead to collective choice of the better of two options, not only in forest tent caterpillars, but also in fish (Ward et al., 2008) and cockroaches (Lihoreau et al., 2010), both group-living animals that forage collectively without a permanent central resting place. This suggests that this mechanism might be widespread among nomadic group-living animals.

The next section explores how responses to social cues also depend on individual temperament and developmental stage.

## Individual traits modulate collective behavior

### Individual variability in responses to social cues

Several species of group-living caterpillars seem to exhibit consistent individual differences in behavior, notably in activity and in exploration, and these differences lead to the emergence of different group-level patterns. For instance, in colonies of *Perga dorsalis* sawflies, certain individuals consistently lead group foraging (Weinstein and Maelzer, 1997). Males of the madrone caterpillar tend to emerge from the nest first and initiate foraging bouts (Underwood and Shapiro, 1999), but in the pine processionary caterpillar, it is females that tend to initiate and lead foraging (Fitzgerald, 2003). In the Eastern tent caterpillar, it has been suggested that different individuals exhibit different thresholds for tasks including silk-spinning, foraging and defensive displaying, and that these differences have a genetic basis (Costa and Ross, 2003). Consistent inter-individual behavioral differences, or animal temperament, have been documented in a wide range of taxa (Sih et al., 2004), and, in social animals, the mix of individual temperaments in groups can determine patterns of collective behavior (Sih and Watters, 2005).

In the Western tent caterpillar, *Malacosoma californicum pluviale*, behavioral types have been postulated to play an important role in colony foraging dynamics. Wellington (1957) showed that certain individuals exhibited independent directed locomotion toward a light source (type I or “active” individuals) whereas others would only advance in the presence of a trail (type II or “sluggish” individuals). Behavioral phenotype was thought to be stable over time, with active individuals arising from the first laid eggs. The proportion of phenotypes varied between colonies and influenced collective dynamics, with active-biased colonies feeding more frequently, building elongated slender tents, exploring a greater volume of the tree crown and building a more extensive trail network, and sluggish-biased colonies building more compact tents and foraging closer to home (Wellington, 1960).

Forest tent caterpillars seem to exhibit a similar difference between active and sluggish individuals: when placed on an unmarked arena in the laboratory, some individuals consistently spent more time exploring the environment, while others were more quiescent (Nemiroff and Despland, 2007). Group-level consequences of these individual behavioral differences were apparent in a nutritionally poor but not in a nutritionally rich environment. When offered a choice between two identical, nutritionally suitable food sources, colonies remained cohesive, foraging together on one of the two food sources. However, when the choice involved two identical unsuitable food sources, colonies containing mostly sluggish individuals remained cohesive, whereas those containing more active individuals fragmented and subgroups foraged simultaneously on the two sources (Dussutour et al., 2008). This environmental effect might explain why several previous studies failed to detect consistent individual differences in behavior (Greenblatt and Witter, 1976; Myers, 1978; Edgerly and Fitzgerald, 1982; Cornell et al., 1988; McClure et al., 2011b).

Thus, it appears that, not only do caterpillars increase exploration and decrease trail following when hungry, as discussed in section “Nutritional environment,” but certain individuals show a greater tendency to do so than others. This suggests that the active and sluggish types first described by Wellington actually reflect differences in trail-following, that is, in responsiveness to social cues. This leads to variation in amplification of initial food choice, whereby high-fidelity trail following (sluggish individuals) implies strong amplification of initial choice and a conservative foraging strategy with the entire colony remaining on that source, but weaker trail-following (i.e., active or protein-deprived individuals) implies more noise, weaker amplification and greater flexibility in foraging, with the colony moving between food sources and occasionally fragmenting. Indeed, a mathematical model shows how this co-existence of two solutions, namely cohesive asymmetric use of a single source and fragmented symmetric use of both sources can arise from differential amplification among individual foragers (Nicolis et al., 2008).

Environmental effects on amplification can generate more conservative patterns in some environments and more flexible ones in others. Wellington (1960) suggested that the adaptive value of the active and sluggish phenotypes should depend on the environment: at low population density, when food quality is high, active colonies should be favored because they feed more often and reach more distant food sources. Indeed, in one study, the more active male biased colonies of madrone caterpillars formed more trails and produced heavier pupae (Underwood and Shapiro, 1999). However, at high population density typical of outbreaks, active colonies risk higher predation rates, greater chance of contact with other colonies and disease transmission, as well as of dispersal into already defoliated regions of the crown (Wellington, 1960). Under these circumstances, the more conservative sluggish colonies should be favored. Similarly, Kause et al. (1999) showed that sawfly larvae active in spring when food quality is heterogeneous within the tree crown disperse to feed, but that those species active in late summer when food quality is uniformly low tend to be more conservative and remain on the same source. In the choice test with two identical nutritionally poor sources mentioned above, the individuals in the active colonies that used both sources grew less during the assay than those in the sluggish colonies (Dussutour et al., 2008). As Wellington (1960) remarked, there is no all-purpose colony; the existence of different behavioral phenotypes, as well as the modulation of individual phenotype by protein satiation, can ensure the emergence of collective foraging behavior appropriate for various environments. The next section examines how caterpillar age also influences responses to social cues and foraging flexibility.

### Ontogenetic shifts in responses to social cues

Many caterpillars exhibit ontogenetic shifts in collective behavior. One common pattern is high cohesion and trail following in early larval stadia, followed by colony break-up later in development, as seen for instance in *Pieris brassicae* (Pieridae) (Long, 1955), *Closyne janais* (Nymphalidae) (Denno and Benrey, 1997), *D. casta* (Reader and Hochuli, 2003), *E. catax* (Ruf et al., 2003), *Anisota senatoria* (Saturniidae), *Symmerista leucitys* and *S. canicosta* (Notodontidae) (Costa, 2006). By contrast, pine processionary (Fabre, 1899) and *Arsenuria armida* (Saturniidae) (Costa et al., 2003) caterpillars are nomadic foragers in the early instars, but later build a permanent nest and switch to central-place foraging.

Forest tent caterpillars exhibit the cohesive collective behavior discussed in the preceding paragraphs in the early instars. However, as the caterpillars mature, their behavior becomes more autonomous. By the fourth larval stadium, caterpillars often forage independently, although they still bask collectively and preferentially follow trails. During the fifth and final larval stadium caterpillars behave mostly independently, though they can still be found in large aggregations during outbreaks when population density is high and many food sources have been depleted. This gradual break-up of colonies emerges from changes in individual responses to social cues, as with the changes in group-level patterns described in sections “Nutritional environment” and “Individual variability in responses to social cues.”

Indeed, as forest tent caterpillars mature, they become more active and less reluctant to deviate from trails. Lab experiments comparing caterpillars in the second and fourth larval stadia show that fourth instar caterpillars spend more time walking and less time searching for trails (Despland and Hamzeh, 2004), are more likely to move in the absence of a trail and more likely to leave a resting colony-mate to initiate foraging (Colasurdo and Despland, 2005). Both second and fourth instar caterpillars travel faster when trails are present, but the difference in speed is much less in the fourth instar larvae (Colasurdo and Despland, 2005).

Similar to the short-term variation based on internal hunger state (section “Nutritional environment”) and the stable differences in temperament (section “Individual variability in responses to social cues”) previously discussed, this gradual ontogenetic weakening in responses to social cues decreases colony cohesion and leads to an increase in collective flexibility in foraging. Indeed, groups of fourth instar caterpillars travel faster to food sources than groups of second instar caterpillars, particularly in the absence of trails. In one experiment only 23% of second instar colonies reached a novel food source within a 4 h assay, whereas 79% of fourth instar colonies did so (Despland and Le Huu, 2007).

The selection pressures responsible for this ontogenetic weakening in responses to social cues are tied to the changes in the advantages to group-living discussed in section “Advantages of collective behavior in the forest tent caterpillar.” In general, larger caterpillars appear to benefit less (and risk more) by living in groups than they did earlier in their development, but the selection pressures involved vary between species (Reavey, 1993): in the tropical *D. casta*, grouped early instar caterpillars benefit from feeding facilitation on tough host plants but larger caterpillars suffer from intraspecific competition (Reader and Hochuli, 2003). In the forest tent caterpillar, as individuals grow larger, predation rates decrease and caterpillars become more able to evade predators (McClure and Despland, 2011) and hence group defense becomes less important. Similarly, older forest tent caterpillars are less dependent on collective thermoregulation, due to both their increased individual thermal mass and higher ambient temperatures as summer advances (McClure and Despland, 2010). In addition, as the season progresses, the probability of disease transmission and of competition for food increases, providing further selection pressures favoring independent locomotion in older caterpillars. Thus, early instar caterpillars grow and develop faster when they are reared in groups, even under laboratory conditions (Robison, 1993; Despland and Le Huu, 2007), whereas under these conditions, grouped older caterpillars experience a decrease in meal length and a reduction in growth, possibly due to interference competition for food (Despland and Le Huu, 2007).

Caterpillars grow larger by several orders of magnitude during development, and, in some species, dispersal seems to be prompted by the associated decrease in predation pressure [e.g., *P. brassicae* (Long, 1955)] or increase in competition for food [e.g., *D. casta* (Reader and Hochuli, 2003) and *C. janais* (Denno and Benrey, 1997)]. However, many species that aggregate early in development and disperse later are early spring feeders [e.g., the New England buckmoth (Stamp and Bowers, 1990b), *E. catax* (Ruf et al., 2003), *Aglais urticae* and *Inachis io* (Nymphalidae) (Bryant et al., 2000)]. These examples suggest that grouping can be a response to the constraints (notably low temperatures) associated with early spring activity, as it lessens as these constraints relax.

## Conclusions

Collective nomadic foraging is common among caterpillars [one study shows 43% of described gregarious caterpillars exhibiting nomadic foraging (Costa and Pierce, 1997)], yet it has received far less attention than central-place foraging. Many nomadic foragers are early spring feeders, and face an environment where food quality is generally high, but ambient temperatures are below optimal. Collective basking is more effective at raising caterpillar body temperature above ambient than is solitary basking and it appears that this is one or even the main advantage to group-living in spring-feeding nomadic foragers. Indeed, the forms of social communication observed in nomadic foragers seem to act mainly in keeping the group together during locomotion between feeding and resting sites.

This high cohesion is necessary for collective basking in the absence of a permanent shelter, but it also implies strong amplification of initial food choices and low collective flexibility as foragers are reluctant to leave the trail and explore new territory. One can therefore hypothesize that nomadic foragers in general should exhibit low selectivity in foraging, as exhibited by the forest tent caterpillar. This might not be a significant disadvantage in an early spring tree crown where food quality is relatively homogenous and high (Hunter and Lechowicz, 1992).

However, the forest tent caterpillar does exhibit some collective flexibility in foraging, via differences in retention according to food source protein content. Indeed, individual protein satiation modulates responses to trail pheromone and hence amplification of the initial choice, with protein-deprived individuals being more likely to leave a trail and seek out novel food sources. However, these individuals are also more likely to be separated from the group, and colony fragmentation is more frequent under nutritional stress, in caterpillars (Fitzgerald and Costa, 1986; Dussutour et al., 2008) as well as vertebrate nomadic foragers (Krause and Ruxton, 2002).

Variation in response threshold to social cues between individuals of different castes or ages is often postulated to direct division of labor in eusocial insect colonies (Beshers et al., 1999), and these differences in response threshold have been linked to concentrations of biogenic amines in the insect brain (Schneider et al., 2006). The present paper shows that modulation of responses to social cues implies changes in individual rules governing interactions between colony-mates, and as the rules change, the emerging group-level patterns also change. Thus, the colony's collective behavior can vary in response to protein richness of environment (via effect of individual internal state on responses to cues), to population density [via an effect of maternal nutrition on the proportion of behavioral phenotypes in a colony (Wellington, 1960)] and to developmental stadium (via ontogenetic shifts in responses to cues).

The findings summarized above suggest that collective behavior in the forest tent caterpillar is driven mainly by the need to stay together to reap the thermal benefits of collective basking in below optimal spring temperatures, but that it is modulated by other factors including protein satiation. The forest tent caterpillar is the best studied nomadic early spring feeder; however, similar forms of social communication appear to exist in other species, as do similar constraints linked to low temperatures and a short phenological window of opportunity, and similar patterns of ontological change (Costa, 2006). The work presented here therefore opens up further avenues of research into the collective behavior of early spring nomadic foragers.

### Conflict of interest statement

The author declares that the research was conducted in the absence of any commercial or financial relationships that could be construed as a potential conflict of interest.
